# The association of eye movements and performance accuracy in a novel sight-reading task

**DOI:** 10.16910/jemr.14.4.5

**Published:** 2021-10-21

**Authors:** Lucas Lörch

**Affiliations:** University of Mannheim, Germany

**Keywords:** Eye movements, musical performance, sight-reading, MIDI data, complex span task

## Abstract

The present study investigated how eye movements were associated with performance accuracy during sight-reading. Participants performed a complex span task in which sequences of single quarter note symbols that either enabled chunking or did not enable chunking were presented for subsequent serial recall. In between the presentation of each note, participants sight-read a notated melody on an electric piano in the tempo of 70 bpm. All melodies were unique but contained four types of note pairs: eighth-eighth, eighthquarter, quarter-eighth, quarter-quarter. Analyses revealed that reading with fewer fixations was associated with a more accurate note onset. Fewer fixations might be advantageous for sight-reading as fewer saccades have to be planned and less information has to be integrated. Moreover, the quarter-quarter note pair was read with a larger number of fixations and the eighth-quarter note pair was read with a longer gaze duration. This suggests that when rhythm is processed, additional beats might trigger re-fixations and unconventional rhythmical patterns might trigger longer gazes. Neither recall accuracy nor chunking processes were found to explain additional variance in the eye movement data.

## Introduction

Sight-reading denotes the performance of a notated melody on a musical instrument without prior practice (26; 49). It involves scanning the musical score with the eyes and translating the perceived symbols into specific movements on a musical instrument. During the scanning of the score, eye movements follow a specific action schema, i.e. they are based on a learned and prototypical sequence of actions (27). Thus, eye movements during sight-reading are not arbitrary but can be considered a highly relevant skill in itself (27). Accordingly, they have received a lot of attention in previous studies (reviewed by Madell & Hébert, 30 and Puurtinen, 40). Amongst others, these studies provided findings how eye movements during sight-reading are associated with musical expertise (2; 17), practice (7; 44), or complexity (18; 29).

However, as discussed by Puurtinen (40), only a small number of studies addressed the role of performance accuracy in the context of sight-reading. This is surprising, as an integration of performance and eye movement measures could help to develop a more comprehensive idea of the sight-reading process. After all, a correct musical performance not a specific way of moving the eyes is the goal during sight-reading. Addressing this issue, the main aim of the present study was to provide insights how eye movements relate to performance accuracy during sight-reading. To this end, I collected and analyzed eye movement and MIDI performance data during a sight-reading task which was embedded in a complex span task (10).

### The assessment of performance accuracy

Taking the studies reviewed by Puurtinen (40) and her considerations on the handling of performance errors as a starting point, I analyzed in detail how performance accuracy was incorporated in previous studies on eye movements during sight-reading. The results of this analysis of the literature can be found in Table 1. It shows past studies on eye movements during sight-reading and (1) how they assessed performance accuracy, (2) which accuracy measures they derived from this assessment and (3) how they used these measures. Besides a number of studies in which it was not reported if or how performance accuracy was assessed (1; 2; 16; 24; 50), I found three main methods of assessing performance accuracy, namely counting errors by hand, using expert ratings, and algorithmic methods.

**Table 1.** Overview of the role of performance accuracy in previous studies on eye movements during sight-reading.

Article	Methods to assess performance accuracy	Measures of performance accuracy	Usage of performance accuracy measures
Goolsby (1994a)	Expert rating	Number of errors	Checking skill differences
Goolsby (1994b)	Expert rating	Number of errors; Musical expression; Musicality	Using errors as complementary information to interpret single cases
Kinsler and Carpenter (1995)	None reported	None reported	None reported
Truitt et al. (1997)	Counting errors by hand	Duration of notes; Position of first error	Analyzing association with size of moving window
Furneaux and Land (1999)	None reported	None reported	None reported
Gilman and Underwood (2003)	Counting errors by hand	Pitch accuracy	Checking skill differences; Analyzing association with size of moving window
Wurtz et al. (2009)	None reported	None reported	None reported
Penttinen and Huovinen (2011)	Counting errors by hand	Number of pitch errors; Deviation of note onset (sixteenth notes)	Checking skill development
Drai-Zerbib et al. (2012)	Algorithm	Number of errors	Analyzing association with fixation duration
Ahken et al. (2012)	None reported	None reported	None reported
Rosemann et al. (2016)	Expert rating	Grade for the quality of the performance	Checking skill development
Penttinen et al. (2015)	Counting errors by hand	Substitution; Addition; Late note	Excluding measurements
Arthur et al. (2016)	None reported	None reported	None reported
Hadley et al. (2018)	Unclear /Algorithm	Pitch deviation (semitones); Deviation of note onset (ms)	Excluding measurements; Descriptive statistics
Huovinen et al. (2018)	Counting errors by hand	Number of errors	Excluding measurements
Cara (2018)	Counting errors by hand	Deletions; Additions; Substitutions; Variability of timing	Creating skill groups; Checking skill differences
Lim et al. (2019)	Algorithm	Rhythmic accuracy; Pitch accuracy; Overall accuracy	Creating skill groups; Analyzing association with eye-hand span
Zhukov et al. (2019)	Expert rating	Score of Watkins-Farnum Performance Scale	Analyzing association with number and duration of fixations
Chitalkina et al. (2021)	Counting errors by hand	Number of errors	Analyzing association with pupil size

Counting of errors by hand is the most prevalent method (7; 8; 17; 22; 37; 38; 48). An output of the recorded MIDI data is produced and a researcher compares this output with the musical stimulus. Errors in pitch and rhythm can be marked in an objective way, as each deviation between the MIDI output and the musical stimulus can be considered an error.

The second method, expert ratings (18, 19; 44; 51), allows to assess performance accuracy even for non-digital instruments that do not produce a MIDI signal. Expert musicians or music researchers listen to an audio recording of the performance and judge its quality according to certain criteria. As this is a rather subjective method, criteria should be clearly defined and reported, the rating should be completed by at least two independent raters and interrater-reliability should be analyzed.

While both these methods might produce valid measures of performance accuracy, their downside is that they are rather time-consuming. For studies that entail large samples with hundreds or even thousands of performances, these approaches are not feasible. In such a case, researchers need to rely on algorithmic solutions. Currently, to my knowledge, there are only three studies that used algorithms to assess performance accuracy during sight-reading (14; 29; 20).

Drai-Zerbib et al. (14) used a Visual Basic program in Excel and Lim et al. (29) used a dynamic time-warping algorithm. In both studies, the algorithms were used to compare the recorded MIDI data from the participants with the MIDI data generated from the stimulus melodies. Lim et al. (29) derived a measure of overall similarity in pitch and rhythm from this comparison. Additionally, the authors used another algorithm, a MIDI-to-MIDI alignment method described by Nakamura, Yoshii, and Katayose (34), to retrieve the number of pitch errors and timing errors separately. The third study using an algorithmic method was the one by Hadley et al. (20). While they did not report their method to assess pitch errors in detail, they compared note onsets of performances with the stimulus score also using a dynamic time warping algorithm.

It becomes clear that there is a need for an easy-to-use program that provides fine-grained measures of performance accuracy for different musical parameters. The present study presents such a program, the *MidiAnalyzer*. It uses the Python programming language (https://www.python.org/) and the package music21 (http://web.mit.edu/music21/). It contains a set of functions explicitly developed to analyze musical performances that resulted from psychological experiments. It can be used without any programming skills and produces binary measures that indicate the correctness of pitch and rhythm on the level of individual notes. Detailed information on the program can be found in the Methods section and in the Appendix.

### The usage of accuracy measures

Past studies mainly used measures of performance accuracy in three ways. First, the studies by Penttinen et al. (38), Hadley et al. (20), and Huovinen et al. (22) used measures of performance accuracy as a criterion to exclude measurements. The authors assumed an association between performance accuracy and eye movements during sight-reading. However, they wanted to focus on other aspects and hence kept performance accuracy on a constant level by excluding inaccurate performances.

Second, a number of studies used performance accuracy as a manipulation check. Penttinen and Huovinen (37) and Rosemann et al. (44) used measures of performance accuracy to check if an assumed improvement of sight-reading skill had occurred after a 9-month music course and after a 30-minute practice period, respectively. In the studies by Goolsby (18), Gilman and Underwood (17), and Cara (7), performance accuracy measures were used to check if the assumed difference in sight-reading skill between expert and non-expert musicians was found.

Third and most interestingly, performance accuracy measures were used in previous studies to test their association with eye movements (8; 14; 29; 51). Chitalkina et al. (8) investigated how local incongruences in familiar music affected the music reading process. The folk song “Mary had a little lamb” and different variations of it were presented to musically experienced participants. The variations had a more complicated tonality and/or one bar was shifted down by two semitones. Participants had to perform the melodies on a piano or had to sing them. Analyses revealed that tn the second half of the altered bar, performance errors were associated with a decrease in pupil size (8).

In the study by Lim et al. (29), simple and complex stimuli were created by varying pitch chromaticism and the number of notes per beat. Both types of melodies were sight-read by musical experts in slow and fast tempo (80 and 104 bpm). The authors found that the size of the eye-hand span was correlated with performance accuracy, with the direction of this correlation depending on the complexity of the stimulus. In simple melodies there was a positive correlation, i.e. more accurate performances were associated with a larger eye-hand span. In complex melodies, on the other hand, there was a negative correlation, i.e. more accurate performances were associated with a smaller eye-hand span. The authors concluded that the eye-hand span is “a strategy that can vary according to the difficulty of the sight-reading task” (29, p. 1).

Drai-Zerbib et al. (14) asked musicians of varying levels of musical expertise to read and then perform musical excerpts. In half of the trials, participants heard the excerpts prior to the first reading. Eye movements were tracked during both initial reading and sight-reading. The authors found that experts with a longer overall gaze duration during the initial reading made more errors during sight-reading. For non-experts, on the other hand, it was found that a longer overall gaze duration during sight-reading was associated with a larger number of errors.

In the study by Zhukov et al. (51), woodwind players performed the sight-reading examples of the Watkins-Farnum Performance Scale. During this task, participants’ eye movements were tracked and their performance was audio recorded. Using these audio recordings, two experts rated the quality of the performances based on the instructions in the Watkins-Farnum Performance Scale. The resulting sight-reading score was negatively related with fixation duration. The authors considered a larger sight-reading score to indicate sight-reading (SR) skills and followed that “players with better SR skills required less time to process musical notation” (51, p. 5).

In summary, while the studies by Chitalkina et al. (8), Lim et al. (29), Drai-Zerbib et al. (14) and Zhukov et al. (51) did report single findings on the association of eye movements with performance accuracy, this association was not the focus of their works. The present study addressed this gap in the literature by providing a first systematic investigation of the association between eye movements and performance accuracy during sight-reading.

I used the number and duration of fixations as central eye movements measures. This decision was based on two arguments. First, information intake happens during fixations. Thus, they are highly relevant for the reading process. Second, they have been widely used in studies on eye movements during sight-reading. Of the 15 studies reviewed by Puurtinen (40), only three (16; 22; 44) did not use the number and/or duration of fixations in their analyses.

### Practice, expertise and features of notes

In order to develop a nuanced understanding of the association between eye movements and performance accuracy, it is highly crucial to consider it in the context of other variables that might affect the reading process. For the present study, I identified three of these variables, namely practice, musical expertise and features of notes.

Practice of a musical piece was found to have a positive effect on performance accuracy (44). Moreover, eye movements were found to change with practice (6; 18). Burman and Booth (6) asked participants of varying levels of sight-reading skill to practice a piece of music according to various rehearsal schedules across several days. After each rehearsal session, participants were asked to complete a perceptual task. In this task, a segment of varying length of the rehearsed piece was presented for 200 ms (called tachistoscopic presentation) with a single note being altered in some trials. Participants had to detect this altered note as quickly and accurately as possible (called error-detection, change-detection or same-different-judgment). Practice moderated the effect of sight-reading skill on the perceptual span. While prior to rehearsal, more skilled sight-readers had a larger perceptual span, this skill differences vanished after 20 rehearsals. In the study by Goolsby (18), participants that were divided in two skill groups performed four different melodies three times each. After the second performance, there was a practice period of four minutes. The author found that the number of fixations decreased and that the duration of fixations increased with repeated encounters of the same melody. However, practice did not moderate the effect of skill level on eye movements, i.e. skill differences remained constant across repeated encounters.

Besides practice, musical expertise was also found to be associated with a more accurate sight-reading performance (6; 7; 14; 18) and with changes in eye movements (2; 18; 38; for a review see Sheridan, Maturi, & Kleinsmith, 47). Gilman and Underwood (17) asked participants of varying expertise to perform three types of tasks, namely a sight-reading task, a transposition task, and an error detection task. In all tasks, notes were presented with a moving window of variable size. This means that only a small area of the score around the point of fixation was visible. Analyses revealed that more experienced musicians read with fewer fixations and with a larger eye-hand span and that they performed the tasks more accurately than less experienced sight-readers.

Lastly, features of notes, such as their tonality, layout or complexity, were found to affect eye movements and performance accuracy (1; 2; 29). Ahken et al. (1) asked expert pianists to sight-read melodies in which the last bar was either congruent or incongruent with melodic expectations of the established tonal context. The melodies were five to seven bars long, used either accidentals or a key signature and there was no control of performance tempo. In melodies with key signature, the authors found the mean fixation duration to increase in the incongruent bar. In the study by Arthur et al. (2) expert and non-expert musicians had to sight-read a four-bar melody and a visually disrupted counterpart in maximal tempo. Visual disruption was created by removing bar lines, altering stem directions, and varying inter-note spacing. The authors reported that when experts read the disrupted score, the saccadic latency increased.

In summary, practice, expertise as well as features of notes can be assumed to affect both eye movements and performance accuracy during sight-reading. Thus, all three of these variables were incorporated in the present study. Participants performed multiple melodies which were created by arranging certain rhythmic fragments in random order and with a quasi-random pitch. Thus, the rhythmic fragments were *practiced* with each performance. In addition, the melodies contained four types of simple note pairs with varying *rhytmical* *features*: eighth-eighth, eighth-quarter, quarter-eighth, quarter-quarter. Eye movements used to read these note pairs were analyzed using areas of interest (AOIs). In the end of the experiment, I collected information on participants’ level of musical *expertise* with the general musical sophistication scale of the Gold-MSI questionnaire (45).

I analyzed if the number of fixations during the reading of the melodies was associated with the performance accuracy measures of the MidiAnalyzer. In addition, I analyzed if the number of fixations and total gaze duration within AOIs was associated with the accuracy of performing the note pairs. In both analyses, the amount of practice and the Gold-MSI score were used as covariates. The type of note pair was used as an additional covariate in the analysis of AOIs. This approach allowed to test if eye movements and performance accuracy were genuinely associated over and above the association potentially caused by one of the covariates.

### Embedding sight-reading in a complex span task

In the present study, a new paradigm was introduced to sight-reading research, namely the working memory complex span task (10). In this type of task, which developed from the reading span task (12), a recall component is combined with a processing component. Participants are typically asked to memorize a memorandum, then process a stimulus, memorize another memorandum, process another stimulus and so on until a serial recall task follows. Commonly, this task is used to analyze working memory processes with the processing task merely functioning as a distractor to prevent rehearsal of memoranda. The present study, however, broke with this convention and used the processing task as the main subject of interest.

In the task used in the present study, single quarter notes of varying pitch were presented as memoranda. The sight-reading of simple, single-staff, four-bar melodies at 70 bpm was used as a processing task. In one half of the trials, sequences of memoranda formed major triads while in the other half, they formed arbitrary trichords. Participants were expected to form memory chunks from major triads, i.e. to store them in a more compressed manner and recall them more accurately (32; 33; 39). I investigated how recall accuracy and chunking processes in the recall task affected eye movements in the sight-reading task.

The time-based resource-sharing theory (4) and the associated computational model TBRS* (36) assume that in complex span tasks, memoranda decay when attention is devoted to the processing task. Hence, it is assumed that memoranda are refreshed frequently. However, this refreshing is assumed to occur only during any *free time*, i.e. in situations were no attention is needed for the processing task. Based on the logic of this theory, I expected that neither the accuracy of recalling notes nor chunking processes would affect eye movements during sight-reading in the present task. If memoranda are refreshed only after the visual information is processed and new saccades are planned, eye movements should not be affected by the refresh processes.

## Research Question

The present study employed a complex span task in which the memorization of single quarter notes for serial recall was alternated with the performance of simple melodies on a piano at first sight. The structure of memoranda was manipulated such that they either formed major triads that supported chunking, or formed arbitrary trichords that did not support chunking. Sight-reading melodies were unique but similar as they all contained four types of note pairs with certain temporal characteristics. The repeated performance of these similar melodies allowed participants to practice the contained musical patterns. Thus, there were three experimental factors, namely *chunking condition*, *practice* and *type of note pair*. I collected four classes of outcome measures: (1) eye movements and (2) performance accuracy during sight-reading, (3) recall accuracy in the serial recall task and (4) musical expertise of the participants.

Using the experimental factors and outcome measures, I investigated the following research question: Are eye movements associated with performance accuracy during sight-reading and does this association prevail when controlling for practice, musical expertise and the type of the processed note pairs? To guarantee that the eye movement measures can be interpreted with respect to this research question, I additionally analyzed if recall accuracy and chunking processes in the recall task explained any additional variance in eye movement measures.

## Method

### Participants

I recruited two groups of participants for the present study. The first group consisted of music students who were recruited at the Mannheim University of Music and Performing Arts. The second group entailed musically literate students who did not study music and who were recruited at the University of Mannheim. Participants of this latter group had to consider themselves to be able to play musical notes on some instrument to participate in the study. The two participant groups will henceforth be called *music students* and *hobby musicians*, respectively. For both groups, participation was not restricted to a particular genre or instrument. Of the initial 155 participants, eleven were excluded due to non-adherence to experimental instructions or missing eye-tracking data, resulting in a final sample size of 144 (*n*_music students_ = 74; *n*_hobby musicians_ = 70). As compensation for taking part in the study, participants either were paid 5 € or received course credits.

I employed the general musical sophistication scale of the Gold-MSI (45) as an indicator of musical expertise. To check if the Gold-MSI score reflected the assumed difference in musical expertise between participant groups, I calculated a one-way ANOVA. It revealed that the Gold-MSI score indeed differed significantly between the groups with music students having a larger Gold-MSI score (*F* (1,142) = 89.49; *p* < .001; *M*_music students_ = 84.54; *SD *_music students_ = 7.06; *M*_hobby musicians_ = 70.83; *SD*_hobby musicians_ = 10.14). A more qualitative understanding of expertise differences between the groups is provided by the single items of the Gold-MSI. Music students indicated to have played an instrument regularly for about ten years (Item 32: *M* = 6.8; *SD* = 0.47; means refer to answering options of the Gold-MSI), to have practiced for three to four hours daily at the height of musical activity (Item 33: *M* = 5.84; *SD* = 1.15), and to be able to play three musical instruments (Item 37: *M* = 4.08; *SD* = 1.18). Hobby musicians, on the other hand, indicated to have played an instrument for four to five years (Item 32: *M *= 5.03; *SD* = 1.73), to have practiced for one hour daily at the height of musical activity (Item 33: *M* = 3.29; *SD* = 1.35), and to be able to play two instruments (Item 37: *M* = 2.78; *SD *= 1.04). Table 2 shows characteristics of the sample.

**Table 2.** Characteristics of the sample of the present study.

Measure	Characteristics
Age	*M* = 22.24; *SD* = 3.87; *Min* = 18; *Max* = 54; 3 missing
Study Subject	Bachelor of Education (Non-Music Subjects): 39 Bachelor of Education (Music): 27 Bachelor of Arts (Music): 28 Master of Arts (Music): 5 Bachelor of Science (Psychology): 25 Others: 10 Missing: 10
Semester	*M* = 4.49; *SD* = 3.3; *Min* = 1; *Max* = 16
Gold-MSI global scale	*M* = 77.88; *SD* = 11.06; *Min* = 46; *Max* = 99
Main instrument	Brass: 9 Keyboard: 34 Percussion: 7 String: 44 Vocals: 12 Woodwind: 32
Sex	84 female; 56 male

### Design and Material

In the present study, I sought to investigate the association of eye movements and performance accuracy during the performance of note pairs with specific temporal characteristics. Accordingly, sight-reading melodies were created based on the within-participants factor *type of note pair* with the four levels eighth-eighth, eighth-quarter, quarter-eighth and quarter-quarter. Based on each note pair, a one-bar rhythmic phrase was created. The note pairs, their temporal characteristics and the associated rhythmic phrases can be found in Table 3. It should be noted that the structure of these phrases was highly similar: the note pair of interest appeared directly after the bar line, allowing a clear identification of its location; the phrases ended with a rest, and contained only fourth and quarter notes and rests. In the analysis of the reading of the note pairs, the factor *type of note pair* was used as a predictor. The temporal characteristics of the note pairs were considered in the interpretation of the effects of this factor.

For the present task, I created four sets of twelve melodies, i.e. 48 melodies overall. All melodies were in treble clef, in a 4/4 meter, contained only notes from the C major scale between C4 and A5 and were four bars long. The rhythm of the melodies was obtained by randomly combining the rhythmic phrases in Table 3 with each phrase appearing once.

**Table 3.** Note pairs and rhythmic phrases used to create sight-reading melodies.

Note pair		Temporal characteristics		Rhythmic phrase
		Total duration	Duration first note	
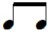	eighth-eighth	1 beat	Short	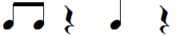
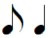	eighth-quarter	1.5 beats	Short	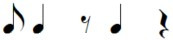
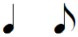	quarter-eighth	1.5 beats	Long	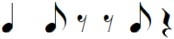
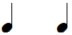	quarter-quarter	2 beats	Long	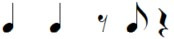

Upon having created the rhythm of the melodies, a pitch had to be chosen for each note. This involved selecting a pitch range for each of the four sets of melodies and assigning a pitch from this pitch range to each note. To select a pitch range, nine candidate pitch ranges were created. These nine candidates started on the notes between C4 and D5 and contained five adjacent notes from the C major scale (i.e. C4-D4-E4-F4-G4; D4-E4-F4-G4-A4; ...; D5-E5-F5-G5-A5). One of these candidate pitch ranges was randomly chosen for each of the four sets of melodies. Then, a pitch was chosen from the respective range of pitches for each note in such a way that no pitch was more than one position away from the previous one. For example, if the randomly chosen range of pitches would have been E4-F4-G4-A4-B4 and the randomly chosen pitch for the first note of a melody would have been F4, the following pitch would have been chosen from E4-F4-G4. By choosing pitches in this fashion, I avoided large intervallic leaps, as they were found to influence eye movements (22).

**Figure 1.**
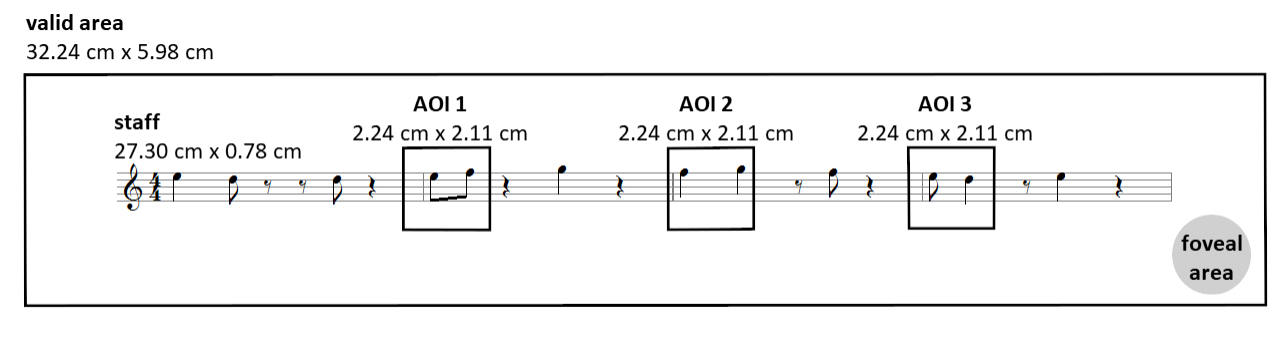
Size of staff and bars and location of AOIs depicted for one exemplary melody.

Overall, the melodies were highly systematic and contained the same elements. However, due to the randomization of the order of rhythmic phrases and the quasi-randomization of pitch, it was impossible for participants to foresee the progression of the melodies and they were forced to actually read and process the notes. Figure 1 shows one example of a sight-reading melody.

Once defined, the melodies were notated with the program Forte 7 Basic (www.fortenotation.com/en/). Using the resulting scores, stimulus images were created with the image manipulation program GIMP (https://www.gimp.org/). The whole staff was 27.3 cm wide (see Figure 1). The clef and meter annotation was 1.3 cm and each bar 6.5 cm wide. The height of the staff was 0.78 cm. One note had the size of 0.2 x 0.7 cm. The staff was inserted in the center of a 49.92 x 28.08 cm (1,920 x 1,080 px) white image. An area with the size of 32.23 x 5.98 cm with the staff at its center was defined as the valid area. All fixations outside this area and the saccades leading to them were discarded. Viewing distance was about 60 cm, hence it could be assumed that an area of 2.1 cm on the screen (2° of the visual field) could be perceived with high acuity (21). The size of this foveal area in relation to the size of the stimulus is depicted in the lower right corner of Figure 1. It should be noted that the layout of the score allowed to perceive each note pair with a single fixation.

AOIs were set in such a way that note pairs could be analyzed in a focused manner (see Figure 1). AOIs had the same position in each melody. As the position of the note pairs varied randomly, AOIs contained different note pairs across melodies. For the analysis, it was coded which AOI contained which type of note pair in which melody. To avoid that differences between note pairs were due to differences in the size of the AOIs, all AOIs had the same size (2.24 x 2.11 cm). In addition, AOIs fulfilled the criteria stated in Holmqvist and Andersson (21). According to these criteria, AOIs should not be smaller than 1.5° visual angle, which is 1.57 cm in the present set-up, with margins of the same size. As will be explained in the Procedure section, there was a short preview of the first bar. Thus, the performance of this bar was no true first sight performance. Hence, there was no AOI around the first note pair.

In order to analyze how chunking processes in the recall task affected eye movements during sight-reading, memoranda were varied on the within-participants factor *chunking condition* with the two levels major triads and arbitrary trichords. In the major triads condition, subsequent memoranda formed major triads, i.e. triads in which the second and third notes had an interval of 4 and 7 semitones to the root note. In the arbitrary trichords condition, subsequent notes formed arbitrary trichords, i.e. trichords in which the second and third notes had an interval of 8 and 9 semitones to the root notes. Major triads were assumed to foster chunking, as they are common and have a clear and meaningful label (such as “C major”). Arbitrary trichords were considered to not foster chunking as they are rather uncommon, are not part of any diatonic major scale and do not have a conventional, meaningful label in any other scale.

In each trial of the task, the pitch of twelve notes had to be recalled. There were two trials in each chunking condition, i.e. four trials overall. To choose the notes of each trial, the root notes, i.e. the notes at serial positions 1, 4, 7, and 10 were chosen randomly from the notes between C4 and D#5. Then, each triad was completed according to the condition. Table 4 shows the memoranda of the four trials, separately for the two conditions.

**Table 4.** Memoranda of the complex span task.

Condition	Memoranda
Major triads	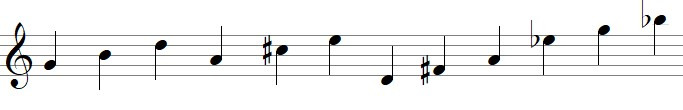
	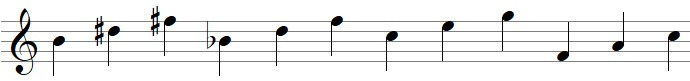
Arbitrary trichords	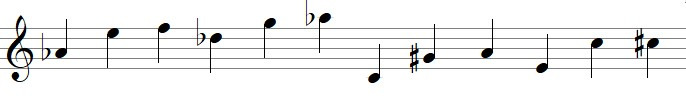
	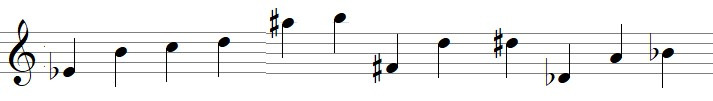

Note. Sight-reading took place in between the presentation of each note.

### Procedure

**Figure 2.**
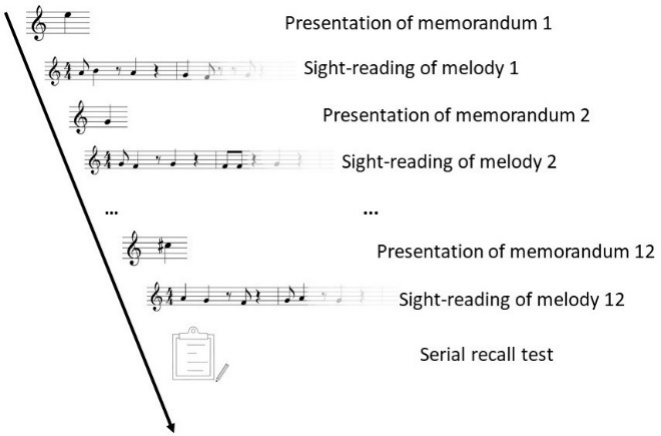
Procedure of one trial of the complex span task.

In the present task, twelve single quarter notes were presented for later serial recall. In between the presentation of each note, participants performed a four-bar notated melody at first sight on an electric piano. The general logic of the task is depicted in Figure 2. Participants saw a note they had to memorize, then had to perform a melody, saw another note they had to memorize, played another melody and so on. After the performance of the twelfth melody, participants had to recall all twelve single notes that they had memorized.

The whole experiment consisted of four phases: (1) instruction, (2) warm-up, (3) complex span task, (4) questionnaires. During the first phase, participants gave informed consent and then were briefed on the upcoming task. They were informed that the task will require them to memorize the pitch of twelve single notes for subsequent serial recall and that they will have to perform a short notated melody on an electric piano together with a metronome between the presentation of each of these notes. These two concurring tasks were communicated as being equally important and unrelated. Then, in the second phase, a warm-up trial followed. It was identical to the following task except for its length: participants had to memorize only three notes and perform only three melodies instead of twelve.

In the complex span task, which was the third phase of the experiment, participants completed the task as shown in Figure 2 four times. This means they played four sets of twelve melodies. The order of the sets was counterbalanced across participants. Prior to each set, there was a preparatory phase, which comprised (a) the positioning of the hand on the piano, (b) an additional preparatory melody for hobby musicians, and (c) the calibration of the eye tracker. Participants were informed how to position their hand in order to play the five tones of the upcoming set. Accordingly, participants were not required to move their hand on the piano during one set of melodies. Then, hobby musicians were provided with a preparatory melody, which consisted of the five tones of the given set, but apart from that was unrelated to the melodies used in the experiment. Hobby musicians were allowed to play this preparatory melody as long as they wanted without metronome in order to learn how the different tones map on the respective piano keys. As a last step in the preparatory phase, the eye tracker was calibrated with a nine-point manual calibration procedure. In the complex span task, the presentation of a memorandum (fixation cross: 2,000 ms; memorandum: 2,500 ms) and the sight-reading of a melody (two bar count-in: 6,857 ms; four bar performance: 13,714 ms) were alternated twelve times as shown in Figure 2. Then, participants were asked to recall the twelve memoranda in the correct serial position on a sheet of paper with an empty staff. Within one set, the whole task was time-controlled without the possibility to stop.

During the sight-reading, the tempo of 70 bpm was provided by a digital metronome via speakers. The performance started after a two bar count-in. During this count-in, participants were provided a preview of the first bar of the melody. Hobby musicians saw this preview for the whole count-in, music students only during the second bar of the count-in. The additional preview as well as the additional preparatory melody for hobby musicians was introduced as I expected the sight-reading task to be demanding for them, especially because only a few of them were pianists. After the count-in, participants had to start to perform the melody and at the same moment, the remaining three bars appeared.

In the last phase of the experiment, participants were asked to complete a number of questionnaires, namely the global scale of the Gold-MSI termed *general musical sophistication* (45), a questionnaire on how they experienced the experiment and on demographics. The experimental procedure was ethically sound with reference to the *Code of Ethics of the World Medical Association* (Declaration of Helsinki); data was treated in accordance with German data privacy regulations (DSGVO).

### Apparatus

Eye movements were recorded during the preview and the performance using a Tobii TX 300 eye tracker (300 Hz sampling rate, 1.0 - 3.3 ms processing latency) connected with a Fujitsu Esprimo P920 desktop computer (intel core i7-4770 3.4 GHz processor, 16 GB RAM, 64 bit operating system). The instructions and the experimental task were presented with the program ePrime 2.0 on the integrated monitor of the eye tracker with a resolution of 1,920 x 1,080 pixels. The digital metronome also was played by ePrime and sounded via Philips SPA 1260/12 speakers. Melodies were performed on a Casio Privia PX-160 electric piano. This piano was connected with a separate Dell Latitude E6330 Laptop (intel core i5-3340M, 2.7 GHz processor, 4 GB RAM, 32 bit operating system) which recorded the Midi signal with the program Cubase Elements 7. No chin rest was used, but participants were instructed to keep their head as steady as possible and the eye tracker was calibrated prior to each set, i.e. four times in the course of the whole experiment.

### Analyses

Fixations were calculated from raw data with the adaptive event detection algorithm by Nyström and Holmqvist (35) with an adaptation for noisy data by Fehringer (15). This algorithm uses the velocity of eye movements to set a threshold for each participant. Eye movements above or below this velocity threshold are defined as saccades and fixations, respectively.

**Figure 3.**
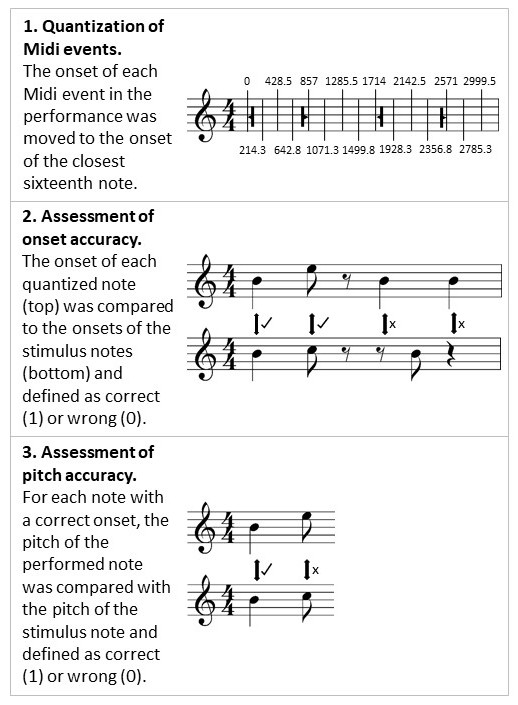
Functionality of the MidiAnalyzer algorithm. Bold lines in the top staff represent Midi events; The numbers above and below the top staff indicate onsets of sixteenth notes in milliseconds.

Participants’ musical performance were analyzed with the MidiAnalyzer algorithm (MidiAnalyze-v1.0; https://doi.org/10.17605/OSF.IO/FKW4B). Figure 3 depicts the three analysis steps performed by this algorithm. As a first step, performances were quantized on sixteenth notes. This means that each note onset was moved to the onset of the closest sixteenth note. With 70 bpm, the fifth sixteenth note of a performance has an onset of 857 ms. If a participant for example would have performed a note with an onset of 840 ms, the quantization would have moved this note to the fifth sixteenth note, i.e. its onset would have been changed to 857 ms. As a second step, the algorithm was programmed to indicate for each quantized note if it was performed at the correct relative position within the melody. This was done by comparing the onsets of the quantized performance with the onsets in the stimulus melody. In addition, the algorithm calculated the mean onset accuracy per melody by dividing the number of performed notes by the number of notes with a correct onset. Lastly, for each performed note with a correct onset, the pitch was compared to the correct pitch. Only notes with correct onsets were considered in this step, as only they had a clear reference pitch. The algorithm derived the mean pitch accuracy per melody by dividing the number of notes with a correct onset by the number of notes that had a correct onset and a correct pitch. In the example in Figure 3, both onset and pitch accuracy would be 0.50. The duration of performed notes was also assessed by the program but was not used in the analysis as participants could not use the piano pedal and thus, it was questionable if this measure provided valid information. A detailed description of the MidiAnalyze algorithm can be found in the Appendix.

To analyze the association of eye movements and performance accuracy, I calculated several mixed linear regression models in R (41) with the package *lme4* (5). Post-hoc analyses were performed with the package *emmeans* (28). The model for the reading of the melodies used the number of fixations as the dependent measure. The duration of fixations was not analyzed. Due to the control of performance tempo, the number of fixations and the mean duration of fixations during the reading of the melodies were negatively related. Thus, analyzing both measures would have been redundant. For the models analyzing the reading of the note pairs, both the gaze duration and the number of fixations within AOIs were used as dependent variables. For these models, the pitch and onset accuracy of performing the note pairs was derived from the onset and pitch accuracy data of the individual notes.

It should be noted that the serial position of the melodies within the sets (i.e. if a melody was the first, second, third, etc. within one trial) was not used as a predictor in the analyses. The reason for this is that serial position confounds two aspects, namely practice and cognitive load. With the performance of each melody of one set, participants were able to practice the contained musical patterns another time but also had to memorize an additional note. The influence of these two aspects could not be separated in the present experiment. Instead, I used the number of sets as an indicator of practice. In each set the musical patterns of the melodies are practiced twelve times. However, as cognitive load increases within each set but not across sets, the variable set does not confounded practice with cognitive load.

For all regression models, following suggestions by Baayen (3), I checked the assumptions of mixed regression, i.e. normal distribution of the residuals, independence of the residuals from the levels of the random factor, homoscedasticity, and normal distribution of the random effects. In addition, I checked multicollinearity of the predictors. Assumptions were fulfilled except for the normality of the random effect. However, mixed regression models were recently shown to provide robust estimates when random effects are non-Gaussian (46).

### Exclusion of measurements

In order to ensure that participants took care in playing the melodies accurately, I checked the mean performance accuracy per participant. To this end, I calculated the mean onset accuracy and pitch accuracy of each participant and then created an average across these two measures. I applied the criterion of mean minus three standard deviations to identify outliers on this variable. No participant fell below this criterion (*M* = 0.74; *SD* = 0.23; *Min* = 0.21). Hence, no participant was excluded due to inaccurate performance.

Concerning the eye-tracking data, first, 8 % of the trials were excluded because either no fixations or no saccades had been tracked. In further 35 % of the trials, the detection of saccades appeared to have been corrupted, as there were either less than three saccades, the tracked saccades spanned less than half the score, or the number of saccades was much smaller than the number of fixations. Fehringer (15) found this problem to occur when the algorithm of Nyström and Holmqvist (35) is used with noisy data. In contrast to standard algorithms that might have problems in the detection of both fixations and saccades when data are noisy, the adaptive approach of Nyström and Holmqvist (35) robustly detects fixations but might miss saccades.

With this in mind, I decided to define two sets of criteria for the exclusion of data points. As fixation measures were the main dependent variables in the regression analyses, the first set of exclusion criteria referred to fixations and was used to exclude whole trials. The second set of criteria referred to saccade measures and was used to remove only single data points, so that implausible values would not affect the reported descriptive statistics.

All exclusion criteria are listed in Table 5. Some of the criteria were chosen based on the frequency distribution of certain variables. For example, the bell curved frequency distribution of the number of fixations had a very long, flat tale to the right, indicating very few measurements of more than 40 fixations. Other criteria were developed based on rational considerations. For example, as the staff was 27 cm wide, an overall distance of progressive saccades of more than 78 cm means that the gaze progressed the whole staff about three times from left to right, which seems unreasonable. After this procedure of excluding data, 5,918 trials, i.e. 86 % of the initial 6,912 trials remained in the data set.

**Table 5.** Criteria for the exclusion of trials and data points.

Exclusion of trials	Trials excluded
Number of fixations < 4	3 %
Number of fixations > 40	1 %
Total gaze duration < 4,571 ms	2 %
Total gaze duration > 13,714 ms	1 %
Fixations outside valid area > 5	2 %
Fixations outside valid area < 0	1 %
Exclusion of data points in saccade measures	Data points removed
Number of saccades < 3	14 %
Distance of forward saccades < 13 cm	30 %
Difference between number of fixations and number of saccades > 8	8 %
Exclusion of measurements in AOIs	Data points removed
Number of fixations > 6	0.7 %
Gaze duration > 4,000 ms	3 %

Note. Total gaze duration denotes the sum of all fixation durations in ms; Overall reading time was 13,714 ms; overall width of the staff was 27.30 cm.

For eye movements within AOIs, there were cases in which no fixation was tracked within an AOI. This resulted either from participants’ gaze skipping an AOI during reading or from measurement noise. Irrespective of the cause, though, these cases did not provide any information for the reading of the note pairs and hence were defined as missing for the analyses. As listed in Table 5, measurements in AOIs with a number of fixations larger than six or a gaze duration larger than 4,000 ms were considered noise and were excluded from further analyses.

In summary, the methodology of the present study followed suggestions by Puurtinen (40) as it collected a large sample of participants, used highly systematic musical stimuli, controlled performance tempo, assessed musical expertise with a standardized questionnaire and analyzed data with multi-level regression models. The dataset, analysis code and experimental material of this study can be found under https://doi.org/10.17605/OSF.IO/9VK57.

## Results

Table 6 shows general descriptive statistics for performance accuracy and eye movements during sight-reading. Overall, participants performed the melodies highly accurately: 68 % of the notes were performed with a correct onset; of these notes with correct onsets, 80 % were performed with correct pitch. Onset and pitch accuracy both were positively correlated with the Gold-MSI score (onset accuracy: *r* = 0.53, *t* (139) = 7.30, *p* < .001; pitch accuracy: *r* = 0.35, *t* (139) = 4.36, *p* < .001). On average, the melodies were read with sixteen fixations that had a duration of about 970 ms. The mean number of regressive saccades (i.e. saccades from right to left) was about four. As one bar was 6.5 cm wide, progressive saccades on average spanned a third of a bar. Regressive saccades had a larger amplitude of about half a bar.

**Table 6.** Means and standard deviations of performance accuracy and eye movements measures during sight-reading.

Measure	Mean	SD
Onset accuracy	0.68	0.32
Pitch accuracy	0.80	0.31
Number of fixations	16.67	7.77
Duration of fixations	971.52	663.13
Number of progressive saccades	12.78	4.94
Number of regressive saccades	4.36	3.26
Distance of progressive saccades	2.28	0.89
Distance of regressive saccades	3.02	2.47

Note. Duration of fixations is in ms; Distance of saccades is in cm.

### The association of eye movements and performance accuracy

Previous studies found first evidence of an association of eye movements with performance accuracy during sight-reading (8; 14; 29; 51). The main goal of the present study was to explore this association in greater detail. To this end, I calculated a mixed linear regression model in which the dependent variable number of fixations was predicted by onset accuracy and pitch accuracy.

As practice (6; 18; 44) and musical expertise (2; 7; 14; 38) were found to influence both performance accuracy as well as eye movements during sight-reading, set and Gold-MSI score were added as covariates in the model. Gold-MSI score and the accuracy variables were z-standardized; set was an integer ranging from zero to three. By-participant random intercepts were added to account for the fact that melodies were nested in participants.

Table 7 shows the parameter estimates of this model. The intercept indicates the estimated number of fixation for a participant with an average Gold-MSI score who performed with average onset and pitch accuracy in the first set. Onset accuracy was the only predictor that was significantly associated with number of fixations. According to the model, an increase of onset accuracy by one standard deviation (i.e. by 32 %) was associated with a decrease of the number of fixations by 0.33. Neither pitch accuracy nor set or Gold-MSI score were significantly associated with number of fixations. The model in Table 7 had a superior fit to the data than a null model without any predictors (*χ*2 (4) = 11.96; p < .05; ΔAIC_initial–null_ = ‑3). This means that the predictors contributed significantly to the explanation of the variance in the variable number of fixations.

**Table 7.** Parameter estimates for the mixed regression model with number of fixations during reading of the melodies as the dependent variable.

Parameter	Estimate	*SE*	*df*	*t*-value
Intercept	16.51	0.53	146	31.12***
Onset accuracy	-0.33	0.14	5711	-2.43*
Pitch accuracy	-0.09	0.09	5632	-1.05
Gold-MSI	-0.82	0.63	143	-1.31
Set	-0.03	0.06	5591	-0.52

*Note:* Gold-MSI, onset accuracy and pitch accuracy were z-standardized. Significance levels * *p* < .05; ** *p* < .01; *** *p* < .001

In summary, this first analysis suggested that, irrespective of musical expertise or practice, melodies that were read with fewer fixations were performed more accurately. This finding can be explained in two ways. First, to sight-read with few fixations might be beneficial for an accurate performance. Planning and executing few saccades saves mental resources for the execution of the notes. Second, performance errors might trigger re-fixations due to the incongruence between the sound that is heard and the sound that is expected.

### Reading and performing the note pairs

Table 8 provides general descriptive statistics for eye movements and performance accuracy within AOIs separately for the four types of note pairs. Descriptively, note onset was less accurate in note pairs starting with an eighth note than in note pairs starting with a quarter note. The quarter-quarter note pair was performed with the most accurate note onset. Pitch accuracy also differed between note pairs in such a way that pitch was less accurate in note pairs starting with a quarter note. Moreover, Table 8 shows that the number of fixations and the number of first-pass fixations within AOIs increased proportionally to the number of beats. The eighth-eighth note pair, which comprised one beat, was read with the smallest number of fixations. The eighth-quarter and quarter-eighth note pair, which both comprised one and a half beats, were read with more fixations than the eighth- eighth note pair. The quarter-quarter note pair, which comprised two beats, was read with the largest number of fixations. Lastly, gaze duration and first-pass gaze duration were markedly increased in the eighth-quarter note pair, but rather similar in the other three note pairs.

**Table 8.** Means and standard deviations of performance accuracy and eye movement measures during reading of note pairs.

	eighth-eighth	eighth-quarter	quarter-eighth	quarter-quarter
Onset accuracy	0.63 (0.44)	0.64 (0.43)	0.69 (0.39)	0.72 (0.39)
Pitch accuracy	0.81 (0.39)	0.81 (0.39)	0.78 (0.41)	0.76 (0.42)
Number of fixations	1.86 (1.06)	1.91 (1.08)	1.93 (1.12)	2.05 (1.16)
Number of first-pass fixations	1.45 (0.76)	1.54 (0.83)	1.55 (0.88)	1.62 (0.90)
Number of second-pass fixations	0.15 (0.45)	0.13 (0.44)	0.14 (0.48)	0.17 (0.50)
Gaze duration	1255.90 (769.88)	1369.80 (772.46)	1214.45 (736.20)	1263.73 (726.21)
Gaze duration of first-pass fixations	1073.00 (754.77)	1201.05 (777.62)	1057.02 (733.43)	1094.93 (729.00)
Gaze duration of second-pass fixations	594.36 (513.21)	601.11 (496.90)	588.46 (494.49)	530.97 (468.37)

Previous studies found that characteristics of notes affected both eye movements as well as performance accuracy during sight-reading (1; 2; 29). Thus, I analyzed how eye movements and performance accuracy were associated during the reading of the note pairs and accounted for the type of the note pair in this analysis. I created a mixed regression model that predicted number of fixation within AOIs by onset accuracy, set, Gold-MSI score, and by the categorical predictor type of note pair. By-melody and by-participant random intercepts were implemented to account for the fact that AOIs were nested in melodies and melodies were nested in participants.

Table 9 shows the parameter estimates for this model. The inclusion of pitch accuracy as a predictor did not increase model fit (*χ*2 (1) = 0.75, *p* = .39). As onset accuracy and Gold-MSI score were z-standardized, as set was an integer ranging from zero to three, and as the eighth-eighth note pair was the reference level, the intercept indicates the estimated number of fixations for a participant with average expertise and onset accuracy in the first set when reading the eighth-eighth note pair. Onset accuracy, the type of note pair and the Gold-MSI score had a significant effect on the number of fixations in AOIs. With an increase of onset accuracy by one standard deviation (i.e. by 41 %), the number of fixations was estimated to decrease by 0.04. Moreover, with an increase of one standard deviation in the Gold-MSI score (i.e. by 11 points), the number of fixations in AOIs was estimated to decrease by 0.10. Post-hoc analysis of the factor type of note pair revealed that the quarter-quarter note pair was read with a larger number of fixations than all other note pairs. The estimated number of fixations in the quarter-quarter note pair was 0.21 larger than in the eighth-eighth note pair, 0.17 larger than in the eighth-quarter note pair and 0.15 larger than in the quarter-eighth note pair. The predictors significantly contributed to the fit of the model (*χ*2 (6) = 74.39, *p* < .001, ΔAIC_initial - null_ = -62).

**Table 9.** Parameter estimates for the mixed regression model with number of fixations in AOIs as the dependent variable.

Parameter	Estimate	*SE*	*df*	*t*-value
Intercept	1.75	0.05	235	37.03***
Onset accuracy	-0.04	0.01	8930	-2.55*
Set	-0.02	0.01	9985	-1.89
Gold-MSI	-0.10	0.05	146	-2.07*
Eighth-quarter	0.04	0.03	2420	1.39
Quarter-eighth	0.06	0.03	3806	2.02*
Quarter-quarter	0.21	0.03	3636	7.33***

Note. Gold-MSI and onset accuracy were z-standardized. Significance levels * *p* < .05; ** *p* < .01; *** *p* < .001

As a next step in the analyses, I modeled the dependent variable total gaze duration within AOIs. The predictors and random effect were the same than the previous model. Table 10 shows the parameter estimates for this model. Again, including pitch accuracy as a predictor did not increase model fit (*χ*2 (1) = 0.87, *p* = .35). All three predictors significantly influenced the gaze duration in AOIs. The model indicated that an increase of onset accuracy by one standard deviation (i.e. by 41 %) was associated with a decrease of gaze duration by 30 milliseconds. Moreover, with an increase of one standard deviation in the Gold-MSI score (i.e. with an increase of 11 points), the gaze duration in AOIs was estimated to decrease by 94 milliseconds. Post-hoc tests of the categorical predictor type of note pair revealed that the eighth-quarter note pair was estimated to be read with a longer gaze duration than all other note pairs. The gaze duration in the eighth-quarter note pair was estimated to be 111 ms longer than in the eighth-eighth note pair, 159 ms longer than in the quarter-eighth note pair and 96 ms longer than in the quarter-quarter note pair. The model fit of the model in Table 10 was superior to the fit of the null model (*χ*2 (6) = 88.81, *p* < .001, ΔAIC_initial - null_ = -77).

**Table 10.** Parameter estimates for the mixed regression model with gaze duration in AOIs as the dependent variable.

Parameter	Estimate	*SE*	*df*	*t*-value
Intercept	1235.61	31.30	240	39.47***
Onset accuracy	-30.87	10.00	8557	-3.09**
Set	-4.91	6.22	9982	-0.79
Gold-MSI	-93.71	32.16	138	-2.91**
Eighth-quarter	110.78	20.20	3261	5.48***
Quarter-eighth	-48.31	20.13	4829	-2.40*
Quarter-quarter	15.23	19.60	4674	0.78

Note. Gold-MSI and onset accuracy were z-standardized. Significance levels * p < .05; ** p < .01; *** p < .001

So in summary, the analysis of AOIs revealed that note pairs that were read with fewer fixations and shorter gazes were performed more accurately. This supports the logic that reading with few fixations is beneficial for an accurate performance. In addition, it appears as if the eighth-quarter note pair was read with a longer gaze duration and the quarter-quarter note pair was read with additional fixations. In the processing of rhythm, unconventional rhythmic pattern (such as the eighth-quarter) might cause longer gazes, while additional beats (as in the quarter-quarter note pair) might trigger additional fixations.

### The association of eye movements with the recall task

Overall, participants recalled 54 % of the notes in the recall task at the correct serial position. To check if the chunking condition had the expected effect on the recall of memoranda, I calculated a one-way ANOVA predicting recall accuracy by chunking condition. The effect of recall accuracy was highly significant and in the expected direction (*F* (1,283) = 27.88; *p* < .001; *M*_major triads_ = 0.65; *SD *_major triads_ = 0.33; *M*_arbitrary trichords_ = 0.45; *SD*_arbitrary trichords_ = 0.30). Thus, it can be assumed that participants indeed build memory chunks from major triads and that this led to a more accurate recall.

To check if eye movements varied with recall accuracy or chunking condition, I included these two variables as additional predictors in the regression models with the dependent variables number of fixations (Table 7), number of fixations in AOIs (Table 9), and gaze duration in AOIs (Table 10). For none of the models, this increased model fit significantly (number of fixations: *χ*2 (2) = 6.18, *p* = .05; number of fixations in AOIs: *χ*2 (2) = 2.92, *p* = .23; gaze duration in AOIs: *χ*2 (2) = 1.02, *p* = .60). This means that neither the more accurate recall of notes nor chunking processes during the complex span task were associated with changes in eye movements.

## Discussion

Previous studies provided initial evidence that eye movements might be associated with performance accuracy during sight-reading (8; 14; 29; 51). The present study took these findings as a starting point and analyzed this association in greater detail. By accounting for the role of practice and musical expertise and by considering both the sight-reading of melodies and of individual notes with specific characteristics, the present study provided a broad perspective on the issue.

Music students and hobby musicians completed a complex span task in which single quarter notes were presented successively for serial recall of pitch. In between the presentation of each note, participants performed a short, notated melody on an electric piano at first sight in the tempo of 70 bpm. Memoranda in this task were manipulated to form either major triads that were assumed to foster the creation of memory chunks, or arbitrary trichords that were assumed to not foster chunking. Sight-reading melodies were created based on four types of note pairs: eighth-eighth, eighth-quarter, quarter-eighth, quarter-quarter. Eye movement and MIDI data were recorded during sight- reading. The MIDI data was analyzed with the newly developed MidiAnalyzer algorithm to derive measures of performance accuracy. The reading and performance of note pairs was analyzed by means of AOIs. Music students had a larger musical expertise score in the Gold-MSI questionnaire than hobby musicians and participants with a larger Gold-MSI score performed the melodies more accurately.

Three mixed linear regression models revealed that (1.1) the number of fixations during the sight-reading of the melodies was negatively associated with the accuracy of note onset, (2.1) the number of fixations during the reading of note pairs was negatively associated with the accuracy of the onset of note pairs, (2.2) the number of fixations was larger for the quarter-quarter note pair than for the other note pairs, (3.1) the total gaze duration during the reading of note pairs was negatively associated with the accuracy of the onset of note pairs, and (3.2) the total gaze duration was longer for the eighth-quarter note pair than for all other note pairs.

As recall was more accurate when memoranda formed major triads, chunking processes seem to have occurred during the recall task. However, neither chunking processes nor recall accuracy explained additional variance in the analyzed eye movement measures.

The finding that the gaze duration on note pairs was negatively associated with the accuracy of performing the note pairs is in line with findings by Drai-Zerbib et al. (14) and Zhukov et al. (51). The former study found that the total gaze duration during the initial reading and during the sight-reading were positively associated with the number of errors during sight-reading. The latter study found that the fixation duration was negatively associated with the sight-reading score. As I will explain in more detail below, longer gazes might indicate a local increase in processing difficulty. This increased processing difficulty might have caused the less accurate performance.

Before introducing a theoretical explanation of the present results, there needs to be a short statement on the issue of causality. The model that analyzed number of fixations during the reading of melodies did not allow to make claims on causal relationships between the variables. In other words, the model did not indicate if certain eye movements caused a certain performance or if a certain performance caused certain eye movements. Number of fixations as well as onset accuracy were outcome measures aggregated across melodies. The matter is more complicated in the analysis of AOIs, though. The point of fixation during music reading is commonly slightly ahead of the point of performance (22; 38; 44). Thus, if a performance error in a note pair would have affected eye movements, these eye movements probably would have been outside the respective AOI. This would consequently not have led to an association of number of fixations and performance accuracy *within AOIs*. Thus, an association of eye movements and performance accuracy within AOIs rather suggests a causal effect of eye movements on performance accuracy.

### On the advantage of reading with few fixations

The main finding of the present study was a negative association of the number of fixations with onset accuracy. This suggests that it might be beneficial for sight-reading to read with few fixations. Reading with few fixations might require less cognitive resources as few saccades have to be planned and less information from different fixations has to be integrated. In contrast, reading with many fixations might rather be seen as chaotic and exploratory. If fixations are poorly timed or placed, they might not provide the information that is required for the performance of the notes. The oculomotor system might search for this information by executing additional fixations, while simultaneously, the lack or delay of information might increase the risk of performance errors.

Although there is stronger evidence for a causal effect of eye movements on performance accuracy, another possible explanation for the present results is that performance errors affected eye movements. Especially expert musicians have been found to be able integrate information across modalities (14). When an error occurs, there is an incongruence between the notes that are read and the music that is heard. This might cause surprise which might trigger a re-fixation. To analyze this in more detail, the MidiAnalyzer could be used in future studies to derive the time points were errors occurred. Thereby, it might be analyzed how the eye movements at these time points were affected by errors.

Furthermore, the present study analyzed only fixation measures and did not find any effects for pitch accuracy. Future studies might test if the association of eye movements and performance accuracy also can be found for other eye movements measures. For example, it might be an interesting question how the number of regressions, pupil dilation or the pupillary-based Index of Cognitive Activity (ICA; 31) are associated with performance accuracy. Moreover, in order to check if the association between eye movements and performance accuracy can be found for pitch accuracy as well, future studies might create sight-reading tasks in which rhythm is constant or quasi-randomized and mainly pitch needs to be processed.

### On the processing of rhythm

In the present study, the type of the note pair had a pronounced influence on both the number of fixations and the gaze duration in AOIs. Not surprisingly, the specific features of the notes that were read seem to have determined the eye movements. The note pairs can be characterized using rhythmical features (duration of the note pair, duration of the first note) or visual features (presence of beams, horizontal distance, similarity of the symbols). Thus, the question arises if the effects should be attributed to the rhythmical or the visual properties of the note pairs.

There is one argument that clearly speaks for referring to rhythmical properties. For both gaze duration and number of fixations, a single note pair differed from all other note pairs. When using visual features, two features are needed to clearly identify these note pairs. In the eighth-quarter note pair, the notes were not similar and had a large horizontal distance. In the quarter-quarter note pair, the notes were similar and had a large horizontal distance. Using the rhythmical properties, though, it is possible to characterize the note pairs with only one feature, namely being uncommon (eighth-quarter) or involving two beats (quarter-quarter). This explanation is more parsimonious and thus should be preferred.

Kinsler and Carpenter (24) likewise claimed that it might not be the visual appearance of notes but rather their meaning that affects eye movements during sight-reading. So what can be learned about the processing of rhythm from the present study? The findings suggest that re-fixations and the prolonging of fixations each might follow their own logic: re-fixations might rather be triggered by additional beats, while the prolonging of fixations might rather be triggered by less common, difficult-to-process rhythmic patterns.

Rhythm processing is a topic that has been largely neglected by sight-reading research. One of the few theoretical accounts was provided by the stochastic model of music reading of Kinsler and Carpenter (24). However, this model was rather complex, was based on the data of merely six participants, and has not received much attention from subsequent research. It might be a more promising approach to first derive single rhythmical features and their association with eye movements before creating a comprehensive theoretical model. The present study went a first step in this pursuit.

### On the meaning of fixation duration during sight-reading

In addition to these implications for rhythm processing, the present results provide insights on the meaning of the duration of fixations during sight-reading. The eighth-quarter note pair was read with longer gazes than all other note pairs. This suggests that fixation duration during sight-reading might be an indicator of local processing difficulty. The eighth-quarter note pair involved a quarter note on the offbeat and hence, was rather uncommon compared to the other three note pairs. This was verbally reported by some participants and by other researchers reviewing the project. Processing such an uncommon rhythmic pattern might have been more difficult which, in turn, might have triggered the longer gaze. The fact that gaze duration was negatively related to performance accuracy also supports this logic. It seems reasonable to assume that notes that are more difficult to process are performed less accurately.

In the domain of text reading, fixation duration is a well-established indicator of processing difficulty (25). Gaze durations during reading have been found to be longer on low-frequency words and unpredictable words than on high-frequency words and predictable words, respectively (13; 23; 42; 43), and shorter when there is a valid preview of the word than when there is no valid preview (9). The effect of word frequency and predictability was also supported in an EEG study by Dambacher and Kliegl (11).

In the sight-reading domain, various factor that can be assumed to increase processing difficulty were found to be associated with longer fixation durations. Longer fixations were found in the sight-reading of musical syntactic incongruities (2) and of more complex musical stimuli (50). In addition, longer fixations were found when experts sight-read visually disrupted scores (1) and when non-experts read larger intervallic skips (37). The studies by Gilman and Underwood (17) and Truitt et al. (48) used a moving window technique, i.e. only parts of the score were visible during reading. A small moving window, which restricted parafoveal preview, was found to be associated with longer fixation durations in both studies.

To further investigate the association of processing difficulty and fixation duration, it would be important to develop objective criteria for processing difficulty in music. The present study suggests that, analogous to the frequency of words in text, the conventionality of rhythms in music might be one criterion. However, it is much more easy to define which words are rare in reading than to define which rhythms are conventional in music, as this depends on the musical background. Just as in text reading, it is possible to conduct corpora studies in music to analyze how often certain rhythmic patterns appear in large bodies of musical pieces. It is unclear, though, if such analyses are useful to derive how common a rhythm is for a musician with a specific background. Alternatively, future studies might ask musicians for their personal opinion how conventional they find certain musical patterns and use these measures as predictors of gaze duration on these patterns during sight-reading.

### On embedding sight-reading in a dual-task

Commonly, in sight-reading experiments, the task is simply to perform melodies at first sight. The present study introduced a new paradigm to sight-reading research, namely the complex span task. During the sight-reading of the melodies, other unrelated notes had to be memorized. The fact that the sight-reading performance was rather accurate implies that participants did not consider the recall task to be the main task but treated both task as equally important just as instructed.

The regression analyses suggest that the recall task did not affect eye movements during sight-reading. Neither for the number of fixation during the reading of the melodies nor for the gaze duration or the number of fixations in AOIs, the recall accuracy or the chunking condition explained any additional variance. This supports the logic of the time-based resource-sharing theory (4) that in complex span tasks, refreshing of memoranda only takes place in free time, i.e. at moments were no cognitive resources are needed for the processing task.

Moreover, self-report questions on how participants memorized the notes indicated that they might have mainly used verbal note names in the recall task. The sight-reading, though, does not involve verbal but rather visuo-motor information. This difference in the formats of the information might have guarded against interference, which might have been another reason for the absence of effects of recall on eye movements.

While using simple sight-reading tasks might be more ecologically valid, this new paradigm provides new opportunities for sight-reading research. Especially for the investigation of expertise, this account seems highly valuable, as it allows a comprehensive perspective on the role of expertise in recall, chunking, sight-reading performance and eye movements. Nevertheless, in order to establish this paradigm as a new tool for sight-reading research, further studies need to replicate the present finding that eye movements are unaffected by recall and chunking processes in complex span tasks.

### On improving the MidiAnalyzer

The MidiAnalyzer has proven to be a practical tool for sight-reading research. It is an open source tool that enables future studies to analyze the accuracy of performances on the level of individual notes fast and objectively. However, there are a number of aspects that might be criticized. First, the MidiAnalyzer only provides binary measures of accuracy. It classifies notes as either being correct or incorrect. How much an incorrect note deviates from a correct note in either pitch or timing is currently not assessed. In addition, the present algorithm indicates pitch accuracy only for notes with correct onsets. The reason for this is that only for a note with a correct onset, it is clear what the reference pitch is. For a note with an incorrect onset, it is unclear if its pitch should be compared with the previous or the following correct note. Nevertheless, especially for statistical analysis, a dependency between variables is never a favorable feature.

**Figure 4.**
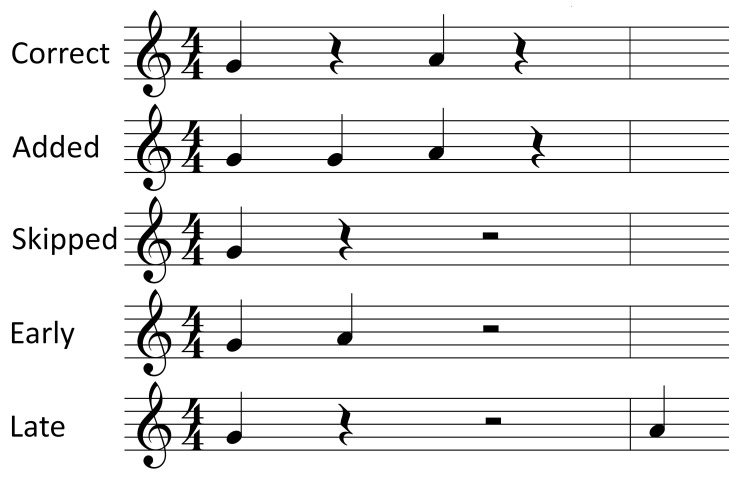
A proposed classification of rhythm errors.

In future versions of the program, different types of rhythm errors might be distinguished. For example, Penttinen et al. (38) distinguished substitutions, additions and late notes, and Cara (7) distinguished deletions, additions and substitutions. Inspired by these works, I developed a slightly different classification. I propose to distinguish four types of rhythm errors, namely added, skipped, early, and late notes. The principle of each type of error is shown in Figure 4.

Added notes are only present when all notes were performed with the correct onset, but some notes were performed in addition. Skips are present when a note is not performed but the previous and the subsequent notes are performed with correct onsets. Lastly, early and late notes are present when there is no note with a correct onset but a note with an earlier or later onset. If there are multiple notes with incorrect onsets, those notes that are closest to the note in question might be defined as early or late notes with all others being defined as added notes.

This classification would not only provide a more detailed view on rhythm errors, it would also allow to provide continuous accuracy measures in some cases. For early and late notes, the deviation of note onset in beats and the deviation of pitch in semitones could be assessed. Thereby, pitch accuracy could also be assessed for notes with incorrect onsets, resolving the dependency between pitch and onset accuracy.

Lastly, the MidiAnalyzer might be extended by specific functions for the integration with eye movement analyses. For example, a future version might contain a function that allows to define AOIs based on some criterion of interest, such as performance errors. This function might be programmed in a way that the output information can be directly plugged into eye tracking algorithms. It would even be possible to create a function that allows to use both MIDI and eye movement data to automatically derive the eye-hand span.

### Conclusion

In summary, the main conclusions of the present study are that (1) sight-reading with few fixations might be beneficial as few saccades have to be planned and less information has to be integrated, (2) performance errors might cause re-fixations due to the incongruence between the expected and the heard sound, (3) in the processing of rhythm, additional beats might trigger re-fixations and unconventional beats might trigger longer gazes, (4) the duration of fixations during sight-reading might indicate local changes in processing difficulty, and (5) when sight-reading is embedded in a complex span task, eye movements might be unaffected by recall accuracy and chunking processes. I hope the present findings and the MidiAnalyzer will spark further interest in the role of performance accuracy in the context of eye movements during sight-reading.

### Ethics and Conflict of Interest

I declare that the contents of the article are in agreement with the ethics described in http://biblio.unibe.ch/portale/elibrary/BOP/jemr/ethics.html and that there is no conflict of interest regarding the publication of this paper.

### Acknowledgements

This work was supported by the University of Mannheim’s Graduate School of Economic and Social Sciences. The publication of this article was funded by the Ministry of Science, Research and the Arts Baden-Württemberg and the University of Mannheim.

I want to thank Benedict Fehringer, Martina Benz, Stefan Münzer, Erkki Huovinen and Elke Lange for their support of this research.

## Appendix

### MidiAnalyze - A Python package for the Analysis of Musical Performance MIDI Data.

**Table:**

Author	Lucas Lörch; LucasLoerch@posteo.de; ORCID https://orcid.org/0000-0002-5665-3313
Download	https://doi.org/10.17605/OSF.IO/FKW4B
License	Creative Commons Attribution-NonCommercial 3.0 Unported License (CC BY-NC 3.0)
Cite	Lörch, L. (2021). MidiAnalyze. A Python Package for the Analysis of Musical Performance Midi Data. https://doi.org/10.17605/OSF.IO/FKW4B

**General idea and purpose**

MidiAnalyze is a Python software package to analyze musical performances on Midi instruments. It has been developed for usage in the context of scientific experiments. The program uses two kinds of Midi files: *stimulus melodies*, i.e. Midi files that hold the notes that participants were asked to perform, and *performances*, i.e. Midi files that hold what the participants played. The functions of the program compare the performances with the stimulus melodies and store the resulting accuracy measures in a spreadsheet.

**Technical specifications**

MidiAnalyze was developed and tested with Python 3.7.1 on Spyder IDE 4.0.0b7 under Windows 10. It has not yet been tested on other operating systems or with other versions of Python. It requires the Python packages *music21*, *sys*, *os*, *glob* and *pandas*.

**Instructions**

1. Prepare software and Midi files

- Install Python and the packages music21, sys, os, glob and pandas. These packages are needed by MidiAnalyze.
- Open the file MidiAnalyze v1-0.py in Python and run the whole code. Now the functions of MidiAnalyze are available.
- Create one folder that contains Midi files of all the original stimulus melodies, i.e. of the melodies participants had to perform during your experiment. These Midi files need to be named according to the item name. For example, if one melody constitutes the first item of condition 1, the filename could be *condition1_item1.mid.* When choosing a name, consider the overall number of conditions and items. If you would have, for example, more than 10 conditions and more than 10 items, you should name your files *condition01_item01.mid*
- Create one folder that contains all the experimental data, i.e. the Midi files that you collected during participants' performances. These midi files need to be named according to the participant identifier and the item name. For example, if participant 15 performed item 1 of condition 1, you could name the file *participant15_condition1_item1.mid.* It is very important that the names of the Midi files holding the original stimulus melodies and the names of the Midi files holding the experimental performances are congruent. Only if this is the case, the stimulus melodies can be assigned correctly to the experimental performances. In the example above, if you would name your experimental file *participant15_condition_1_item_1.mid*, the program would not be able to assign the stimulus melody *condition1_item1.mid* to it, as the item identifier differs. Moreover, it is important that your Midi files contain only one performance and that this performance is aligned with the beat. In some experiments, one might just let the recording running during the whole experiment, comprising the performance of multiple stimulus melodies. If this is the case, it is necessary to use some software that is able to edit Midi and to extract the single melodies and export them as Midi files.

2. Import the Midi files to MidiAnalyze

Run the command getyourMidiFiles(performanceDataPath, solutionDataPath, startItemName, endItemName, startSubjectName, endSubjectName) in Python. As *perfomanceDataPath*, enter the path to the folder with the performance data in quotation marks. Note that Python requires double-backslashes in filepaths under Windows. For example, if your performance data is in a folder "Experiment" on the desktop, the path would be "C:\\Users\\YourName\\Desktop\\Experiment\\". As *solutionDataPath* enter the path to the folder with the original stimulus melodies in quotation marks. As *startItemName* and *endItemName*, enter the position at which the item identifier starts and ends in the file names of your performance Midi files. Note that in Python, the first position is indicated by 0. For example, if your file name would be “participant15_condition1_item1.mid”, the item identifier (which is “condition1_item1”) starts at the 14th position (at the c) so startItemName would be 14. The identifier ends at the 30th position (at the .) so endItemName would be 30. As *startSubjectName* and *endSubjectName* enter the position at which the participant identifier starts and ends in the filenames of your performance Midi files in the same manner. So the full command could for example read getYourMidiFiles("C:\\Users\\YourName\\Desktop\\Experiment\\", "C:\\Users\\YourName\\Desktop\\Stimuli\\", 14, 30, 0, 13)
If needed, all Midi files can be quantized by running the command quantizeMidi(noteValue, quantizeOffsets, quantizeDurations) in Python. As *noteValue*, indicate on which note value the quantization should be based (1=quarter, 2=eighth, 4=sixteenth, 0.5=half, 0.25=whole, 3=eighth triplets). Set the number in square brackets. If *quantizeOffsets* is set to TRUE, the beginning of each note is quantized. If *quantizeDurations* is set to TRUE, the end of each note is quantized. The quantization moves the beginning and/or the duration of each note to the closest note with the value specified. For example, if the specified note value is eighth note and quantizeOffsets and quantizeDurations is set to TRUE, both the beginning and the end of each note is moved to the closest eighth note. The whole command in this case would be quantizeMidi([2], TRUE, TRUE). Note that the Midi information is only changed internally. The Midi files themselves are not affected by the quantization.

3. Analyze the performances and export the resulting spreadsheet

Run the command analyzeMidi() in Python. MidiAnalyze calculates the accuracy measures and descriptives for all performance files and stores them in a spreadsheet.
Run the command exportResults(resultsFileName) in Python. As *resultsFileName*, indicate how you want to name the file that will be generated. Use quotation marks. MidiAnalyze then exports all results as a .csv spreadsheet in the folder with the original stimulus melodies. The full command could for example read exportResults(“MyExperiment_results”). Then the file MyExperiment_results.csv would be created in "C:/Users/YourName/Desktop/Stimuli".

**Functions**

*Utility functions*

These functions handle the steps necessary to perform the analysis. They import the Midi files and create or export the resulting spreadsheet.

**Table:**

Function	Description
indicateFilePaths(“performanceDataPath”, “solutionDataPath”)	Checks and defines the two central file paths, i.e., the performanceDataPath which is the path to the Midi files that resulted from participants’ performance and the solutionDataPath which is the path to the Midi files that hold the stimulus melodies.
importMidiFiles(“performanceDataPath”, startItemName, endItemName, startSubjectName, endSubjectName)	Imports all performance Midi files. Creates a spreadsheet with a row for each file. If the filenames of the Midi files contain item and participant identifiers the location of these identifiers in the filename can be indicated and then, these identifiers are also added to the spreadsheet. Creates the columns *MidiFile*, *item*, *participant* in the spreadsheet.
importCorrectSolutions(“solutionDataPath”)	Imports all stimulus melodies.
addCorrectSolutions()	Matches the stimulus melodies to the performances. Works only if the item identifier is identical in filenames of stimulus melodies and performances. Creates the column *correct* in the spreadsheet.
quantizeMidi([noteValue], quantizeOffsets, quantizeDurations)	Quantizes the performances. If quantizeOffsets is set to TRUE, the beginning of each note is quantized. If quantizeDurations is set to TRUE, the end of each note is quantized. The quantization value is specified by noteValue (1=quarter notes, 2=eighth, 4=sixteenth, 0.5=half, 0.25=whole, ect.). Beginning and/or end of the note are moved to the closest note value.
exportResults()	Creates a .csv file from the spreadsheet and stores it in the folder with the stimulus melodies.

*Compare functions*

These functions compare the performances with the stimulus melodies on three main parameters: the beginning (also called position), pitch and duration (also called note value) of the notes. These functions are the main part of the program as they provide an analysis of performance accuracy.

**Table:**

Function	Description
compareNumberOfNotes()	Creates a column *ommissionAddition* and compares the number of performed notes with the number of notes in the stimulus melodies. 0 means that the correct number of notes was performed. -3 means that the performance contained three notes less than the stimulus melody. +1 means that the performance contained one note more than the stimulus melodies.
compareNotePositions()	Compares the onset of each note in each performance with the stimulus melody and stores a relative accuracy value in the column *ACC_notePosition* (0.5 means that 50% of the performed notes started at the correct position)
compareNotePositionsOfSingleNotes()	For each note X in each stimulus melody, a variable *ACC_notePosition_noteX* is created in the spreadsheet. For each performance, the program indicates if it contains a note that starts at this positions (1) or not (0).
comparePitch()	Compares the pitch of each note in each performance that starts at a correct position and stores a relative accuracy value in the column *ACC_pitch* (0.5 means that 50% of the notes that started at the correct position had a correct pitch). The octave is not considered, i.e. if C4 had to be played and C5 was played, this counted as correct.
comparePitchWithOctave()	Same as comparePitch, but takes octaves into account, i.e. if C4 had to be played and C5 was played, this is counted as wrong. Stores a relative accuracy value in the column *ACC_pitchWithOctave*
comparePitchOfSingleNotes()	For each note Y in each performance that started at a correct position, a variable *ACC_pitch_noteY* is created in the spreadsheet. The program indicates if this note had a correct pitch (1) or not (0).
compareDuration()	Compares the duration of each note in the performances that starts at a correct position and stores a relative accuracy value in the column *ACC_duration* (0.5 means that 50% of the notes that started at the correct position had a correct duration).
compareDurationOfSingleNotes()	For each note Z in each performance that starts at a correct position, a variable *ACC_duration_noteZ* is created in the spreadsheet. The program indicates if this note had a correct duration (1) or not (0).

*Describe functions*

These functions do not compare, but describe the performances. This can be especially useful to check the validity of the performance Midi files by checking if certain measures such as the length of the performance or the pitch range take plausible values.

**Table:**

Function	Description
describeNumberOfNotes()	Creates a column *numberOfPerformedNotes* in the spreadsheet and indicates the number of notes in the performances.
describeNumberOfChords()	Creates a column *numberOfPerformedChords* in the spreadsheet and indicates the number of chords (simultaneously performed notes) in the performances.
describePitchSpan()	Creates a column *pitchRange* in the spreadsheet and indicates the lowest and highest pitches of the performances.
describeNoteValues()	Creates a column *performedNoteValues* and indicates a list of which note values were contained in the performances.
describeDuration()	Creates a column *durationOfPerformanceInQuarterNotes* and indicates the duration of the performances in quarter notes.
describeNumberOfBars()	Creates a column *numberOfPerformedBars* and indicates the length of the performances in bars. Works only if the Midi files contain bar markers.
describeMeter()	Creates a column *meter* and indicates the meter of the performances.
describeInstrument()	Creates a column *instrument* and indicates the instrument information of the Midi files.
describeClef()	Creates a column *clef* and indicates the clef of the performances.
describeTempo()	Creates a column *tempo* and indicates the tempo of the performances
describeKey()	Creates a column *key* and indicates the key of the performances.
describePerformedNotes()	Creates a column *performedNotes* that contains the whole performance in the format [{Note1: [{offset: 0}, {pitch:A4}, {notevalue:0.5}]},{Note2:...}] So for each note, its beginning (offset), its pitch and its duration (notevalue) is indicated.

*Comprehensive functions*

These functions integrate several of the previous function in order to support usability. They do not provide anything new, but only allow to call sets of functions with one command. In principle, all functions of the program can be called with the getYourMidiFiles and the analyzeMidi functions.

**Table:**

Function	Description
getYourMidiFiles (performanceDataPath, solutionDataPath, startItemName, endItemName, startSubjectName, endSubjectName)	Calls the functions indicateFilePaths, importMidiFiles, importCorrectSolutions and addCorrectSolutions.
compareMidi()	Calls the functions compareNumberOfNotes, compareNotePositions, comparePitch and compareDuration
describeMidi()	Calls the functions describeNumberOfNotes(), describePitchSpan(), describeNoteValues(), describeDuration(), and describePerformedNotes(). If the performance Midi files contain information on instrument, clef, tempo, key, bars and if they contain chords, the respective describe functions are called.
analyzeMidi()	Calls both the describeMidi and the compareMidi functions
